# A scoping review to evaluate the efficacy of combining traditional healing and modern psychiatry in global mental healthcare – CORRIGENDUM

**DOI:** 10.1017/gmh.2025.32

**Published:** 2025-03-27

**Authors:** Sagar Jilka, Catherine Winsper, Samantha A. Johnson, Onaedo Ilozumba, Ryan G Wagner, Sanjana Subhedar, Dafne Morroni, Richard Lilford, Swaran P. Singh

Upon publication of this article the affiliation ‘MRC/Wits Agincourt Research Unit, School of Public Health, Faculty of Health Sciences, University of Witwatersrand, Johannesburg, South Africa’ was incorrectly listed against author Swaran Singh. This should not have been included in his affiliation list.

The full author and affiliation list is

Sagar Jilka^1,2,3^, Catherine Winsper^1^, Samantha A. Johnson^4^, Onaedo Ilozumba^5^, Ryan G Wagner^6^, Sanjana Subhedar^1^, Dafne Morroni^1^, Richard Lilford^5^, Swaran P. Singh^1,3^ and On behalf of the TRANSFORM consortium^*^

^1^Warwick Medical School, University of Warwick, Coventry, UK;

^2^Institute of Psychiatry, Psychology and Neuroscience, King’s College London, London, UK;

^3^Warwick Centre for Global Health, University of Warwick, Coventry, UK;

^4^Library Services, University of Warwick, Coventry, UK;

^5^Institute of Applied Health Research, University of Birmingham, Birmingham, UK;

^6^MRC/Wits Agincourt Research Unit, School of Public Health, Faculty of Health Sciences, University of Witwatersrand, Johannesburg, South Africa

^*^The Transforming access to care for serious mental disorders in slums; https://warwick.ac.uk/fac/sci/med/research/hscience/mhwellbeing/projects/transform

The article has been updated.

## References

[r1] Jilka S, Winsper C, Johnson SA, Ilozumba O, Wagner RG, Subhedar S, Morroni D, Lilford R, Singh SP and On behalf of the TRANSFORM consortium (2025) A scoping review to evaluate the efficacy of combining traditional healing and modern psychiatry in global mental healthcare. Cambridge Prisms: Global Mental Health 12, e35, 1–12. 10.1017/gmh.2025.20 PMC11949735 40160384

