# Supplementary material for: A scoping review to evaluate the efficacy of combining traditional healing and modern psychiatry in global mental healthcare
Source: Glob Ment Health (Camb). 2025 Feb 27;12:e35. doi: 10.1017/gmh.2025.20 (PMC11949735; doi:10.1017/gmh.2025.20)
Supplement: Jilka et al. supplementary material [file S2054425125000202sup001.docx]

**Supplementary Materials**

| **Supplementary Table 1. PRISMA-ScR Checklist** | | |
| --- | --- | --- |
| Section | Item | PRISMA-ScR Checklist Item |
| **Title** | 1 | Report identified as a scoping review |
| **Abstract**  Structured summary | 2 | Structured abstract including background, aims, sources of evidence, methods, results and conclusions (relating to research questions): page 2  Eligibility criteria described in methods due to space limitations: page 3 |
| **Introduction** |  |  |
| Rationale | 3 | Rationale for review described with reference to extant literature: page 3, paragraphs 3 and 4  Explanation of why scoping review suitable included in the methods section: page 3, last paragraph. |
| Objectives | 4 | Research questions defined explicitly including reference to their key elements: page 3, paragraph 5 |
| **Methods** |  |  |
| Protocol and registration | 5 | Protocol not registered. |
| Eligibility criteria | 6 | Eligibility criteria included publication status, study design, characteristics of interventions considered, populations of interest, and language constraints: page 3 |
| Information sources | 7 | Information sources described (including dates of all searches): page 4, third paragraph |
| Search | 8 | Example of full electronic search strategy included in Supplementary Table 2 |
| Selection of sources of evidence | 9 | Included top of page 4 |
| Data charting process | 10 | Described bottom of page 4 |
| Data items | 11 | See Tables 1 and 2 |
| Critical appraisal of individual sources of evidence | 12 | Optional: Not done |
| Summary measures | 13 | Not applicable for scoping reviews |
| Synthesis of results | 14 | Described top of page 5 |
| Risk of bias across studies | 15 | Not applicable for scoping reviews |
| Additional analysis | 16 | Not applicable for scoping reviews |
| **Results** |  |  |
| Selection of sources of evidence | 17 | Included page 5, third paragraph and Figure 1 (PRISMA flow chart) |
| Characteristics of sources of evidence | 18 | Studies characteristics and citations provided: page 5 |
| Critical appraisal of sources of evidence | 19 | Optional: Not done |
| Results of individual sources of evidence | 20 | Included in tables 1 and 2 |
| Synthesis of results | 21 | Results were synthesised in text and Tables 1 and 2 pertaining to the research questions: (1) types of available evidence; (2) typologies of collaboration; (3) outcomes of collaborations; and 4) potential mechanisms and contextual moderators. |
| Risk of bias across studies | 22 | Not applicable for scoping reviews |
| Additional analysis | 23 | Not applicable for scoping reviews |
| **Discussion** |  |  |
| Summary of evidence | 24 | Summary of main results provided: top of page 8 |
| Limitations | 25 | Limitations of the scoping review approach: top of page 9 |
| Conclusions | 26 | Main results in relation to research questions: page 8  Implications of results: page 9 |
| **Funding** | 27 | This review is funded by the UK's National Institute for Health and Care Research (NIHR) (Award number: NIHR200846). S.P.S. is supported by the NIHR Applied Research Collaboration (ARC) West Midlands. The views expressed are those of the author(s) and not necessarily those of the NIHR or the Department of Health and Social Care.  Funding for the individual studies:  ^1^ Adelekan 2001: West African Health Community (WAHC/RG/91/001)  ^2^ Gureje 2020: US National Institute of Mental Health.  ^3^ Lam 2016: None  ^4^ Ofori-Atta 2018: Funded by a grant from the University of Ghana and an Unrestricted Grant from Enterprise Insurance Ghana Ltd. Clinical Staff and medications were provided by the Ghana Health Service.  ^5^ Saha 2019/Shields 2016: Nil  ^6^ Veling 2018: National Institute of Mental Health under award R21MH93298  ^7^ Yaro 2020: DFID grant |

**Supplementary Table 2. Electronic search strategy conducted in MEDLINE (OVID)**

| **#** | **Query** |
| --- | --- |
| 1 | exp Medicine, Traditional/ or faith healers.mp. or exp Faith Healing/ |
| 2 | exp Psychiatry/ or psychiat*.mp. |
| 3 | doctor*.mp. or exp Physicians/ |
| 4 | 2 or 3 |
| 5 | 1 and 4 |
| 6 | mental disorders/ or mood disorders/ or "schizophrenia spectrum and other psychotic disorders"/ |
| 7 | mental illness*.mp. |
| 8 | 6 or 7 |
| 9 | 5 and 8 |
